# Real-Time Intelligent Detection Algorithm for Ship Targets in High-Resolution Wide-Swath Sea Surface Images Captured by Airborne Cameras

**DOI:** 10.3390/s26061786

**Published:** 2026-03-12

**Authors:** Haiying Liu, Qiang Fu, Haoyu Wang, Huaide Zhou, Yingchao Li, Huilin Jiang

**Affiliations:** 1 School of Optoelectronic Engineering, Changchun University of Science and Technology, Changchun 130022, China; 2 Jilin Dongguang Group Co., Ltd., Changchun 130103, China; 3 Changchun Institute of Optics, Fine Mechanics and Physics, Chinese Academy of Sciences, Changchun 130033, China

**Keywords:** ship detection, wide-format aerial camera, lightweight YOLOv8, embedded system

## Abstract

**Highlights:**

**What are the main findings?**

- The proposed lightweight YOLOv8 model, enhanced with a Multi-Scale Fusion (MSF) module and Group-Wise Scale Fusion Neck (GSF-Neck), achieves a high mAP@0.5 of 94.55% for ship detection in aerial imagery, significantly outperforming baseline and mainstream detectors. Compared with the baseline under identical conditions, the proposed model improves mAP by 1.4% with only a 6.6% decrease in FPS, achieving a balanced trade-off between detection accuracy and computational efficiency.
- The optimized model effectively processes single large-format aerial images (≥300 MB) in real time at ≥2 fps on an RK3588 embedded system, with a detection rate of ≥89.5% in aerial tests.

**What are the implications of the main findings?**

- This work proposes a highly efficient and practical embedded vision solution that enables real-time ship detection on aerial platforms for maritime surveillance, thereby eliminating the need for cloud processing.
- Moreover, the MSF and GSF-Neck modules introduced in this study provide a reproducible design framework to balance multi-level feature extraction and computational efficiency, which can be extended to detect other resource-constrained objects.

**Abstract:**

The critical task of ship detection in aerial imagery for maritime monitoring faces significant challenges in achieving real-time performance on embedded platforms. These challenges arise from the large data volume inherent in wide-format aerial images and the pronounced scale variations among vessels. To address this issue, an optimized YOLOv8-based model is proposed. Scale adaptability is enhanced by incorporating a Multi-Scale Fusion (MSF) module into the backbone. In addition, a lightweight Group-Wise Scale Fusion Neck (GSF-Neck) with a parallel multi-branch structure is designed to facilitate adaptive multi-scale feature fusion while reducing computational overhead. The proposed model achieves a state-of-the-art mAP@0.5 of up to 94.55% on a dedicated aerial ship dataset, outperforming other major detectors. When deployed on an RK3588 embedded system using a sliding window strategy to process single 300 MB images, it maintains a stable processing speed of ≥2 fps. Compared to the baseline under identical conditions, the model proposed in this study improves mAP by 1.4% with a 6.6% reduction in FPS, effectively balancing detection performance and computational efficiency.

## 1. Introduction

Ship detection on aerial platforms plays an important role in various applications, such as national defense security, fishery management, navigation safety, and maritime search and rescue [1]. With the rapid development of aerial remote sensing technology, wide-format cameras mounted on manned aircraft and unmanned aerial vehicles (UAVs) have become a key approach for maritime surveillance [2]. Nevertheless, ship detection under complex marine environmental conditions remains challenging. Factors such as wave clutter, background interference, and varying illumination often lead to unsatisfactory detection performance. In addition, the excessive data volume of single wide-format images poses a substantial challenge to the real-time processing capability of embedded platforms [3]. Furthermore, bandwidth and latency issues restrict cloud-based processing solutions; therefore, they are unsuitable for time-critical scenarios such as emergency response and battlefield reconnaissance [4].

Deep learning technology has opened a new avenue for real-time ship detection [5]. Existing deep learning-based detectors are generally classified into single-stage and two-stage categories. Two-stage detectors, such as Faster R-CNN [6], offer high accuracy but suffer from low inference speed and high computational cost, which limits their application on resource-constrained aerial platforms [7]. In contrast, single-stage detectors, including the YOLO series [8,9], adopt an end-to-end regression strategy to achieve a better balance between speed and accuracy, making them suitable for real-time tasks.

Standard single-stage models such as YOLOv8 [9] demonstrate high efficiency; however, their application to large-format aerial imagery remains challenging. They encounter significant scale variations in maritime targets, ranging from small boats to large vessels, along with a high computational burden when directly deployed on embedded platforms [10,11]. Therefore, for embedded real-time aerial maritime detection, achieving an optimal trade-off between multi-level feature representation capability and computational efficiency is a key research objective.

To address this issue, the present study proposes a structurally re-engineered YOLOv8 model. The main contributions are as follows: (1) a Multi-Scale Fusion (MSF) module is integrated into the backbone network to improve scale adaptability by effectively capturing features from objects of widely varying sizes. (2) A lightweight Group-wise Scale Fusion Neck (GSF-Neck) incorporating GSConv [11] and GSCSP modules is designed to significantly reduce computational complexity while preserving rich feature representation. The optimized model is deployed on an RK3588 embedded system, demonstrating a practical balance for high-speed processing applications.

## 2. Aerial Camera Platform Systems

### 2.1. System Composition

**Figure 1:**
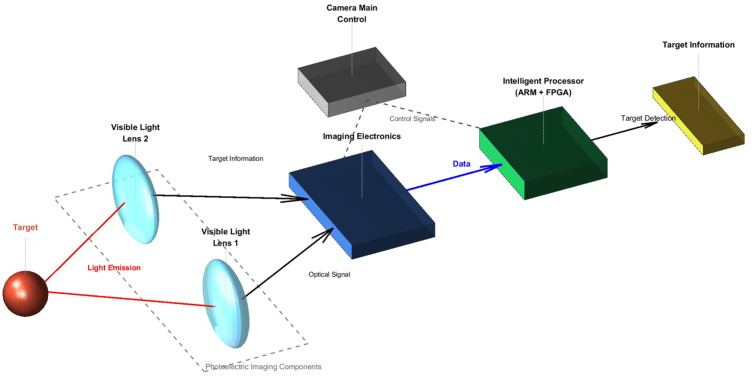
System composition.

The system consists of optoelectronic imaging components (visible light lens and imaging electronics), an intelligent data processing module, and other auxiliary components. The system composition is illustrated in Figure 1.

The intelligent data processing module employs an RK3588J [12] manufactured by Rockchip Electronics Co., Ltd. (Fuzhou, Fujian, China), an ARM processor (an Advanced RISC Machine processor for general-purpose computing) with an octa-core 64-bit architecture (4 × Cortex-A76 + 4 × Cortex-A55), a CPU main frequency of 2.2 GHz, and a GPU main frequency of 1 GHz. 

The CMOS detector used in the optoelectronic imaging component has a package size of 38.75 × 27.75 mm, a pixel size of 4.6 μm, and an image plane resolution of 8424 × 6032 pixels. To further expand the field-of-view angle, optical and structural designs are implemented to splice four imaging devices. Through this design, a spliced field-of-view angle of 62° is achieved, with a coverage width of 6 km (altitude of 5000 m) and a pixel resolution of ≤0.19 m (altitude of 5000 m) for visible light imaging. The data volume of a single image reaches 300 MB, and the image acquisition cycle time is 0.45 s. To enhance target detail and improve real-time processing efficiency in reconnaissance applications, the system outputs grayscale images. The design parameters of the photoelectric imaging system are listed in Table 1.

### 2.2. Platform Working Principle

The system controls the photoelectric imaging module to capture images according to the input photography parameters. Ground scene light passes through the two lens windows of the photoelectric imaging module and is incident on the corresponding photographic objective lens groups. Through transmission and splicing reflector spectroscopy, the light is imaged onto the photosensitive surfaces of four CMOS components. After photoelectric conversion by the detector, the optical imaging information of ground targets is transformed into digital image data. The FPGA module receives the image data, controls the photoelectric imaging module to capture images according to the photographic cycle, synchronizes the imaging data, and caches the synchronized image data into DDR (double data rate synchronous dynamic random-access memory). Subsequently, operations such as grayscale consistency adjustment, automatic dimming, and image splicing are performed based on image brightness information. The FPGA processor receives image read commands from the ARM processor and transmits the cached images to the ARM processor for target detection using the improved YOLOv8 algorithm so as to determine whether valuable targets exist in images from different scenes. If a target is detected, the detection result is sent back to the FPGA, which calibrates the result and then outputs the image.

## 3. Selection of the YOLOv8 Model

**Figure 2:**
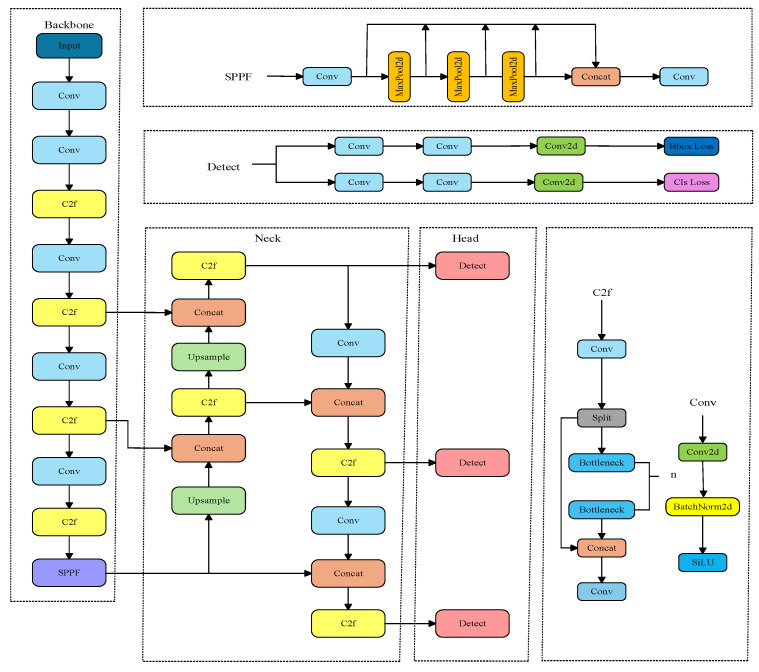
YOLOv8 network architecture.

After a comprehensive evaluation, Ultralytics YOLOv8 [13] was selected as the baseline model in this study due to its balanced performance, flexibility, and extensive community support, which facilitate model implementation and modification. The network architecture of YOLOv8 is illustrated in Figure 2. It integrates a Feature Pyramid Network (FPN) and a Path Aggregation Network (PAN) and mainly consists of a backbone, neck, and head. The FPN propagates deep semantic features through a bottom-up pathway and fuses them with shallow features to enhance multi-scale target representation. The PAN further complements this process by reintroducing detailed shallow features into deeper layers through a top-down pathway, effectively mitigating information loss in deep feature maps. This bidirectional multi-scale fusion mechanism efficiently combines deep semantic information with shallow spatial details, enabling the network to better handle features and semantics at different scales.

YOLOv8 provides five pre-trained models of different scales: n, s, m, l, and x. Their object detection performance is summarized in Table 2. Considering the strict real-time requirements of maritime target detection, the YOLOv8-s model was selected in this work.

The selection of YOLOv8-s as the baseline model represents an optimal choice based on comprehensive consideration of three core factors: the requirements of large-format maritime image detection, the resource constraints of embedded platforms, and the balance between model performance and computational cost. Further details of the rationale are provided below:

Real-Time Processing Requirement: To satisfy stringent real-time requirements, the embedded platform should process individual ultra-large images (≥300 MB) at ≥2 frames per second. This is achieved by dividing each image into 330 tiles of 640 × 640 pixels for parallel inference. Although large models (YOLOv8-m/l/x) exhibit improved accuracy, their per-tile inference times are significantly longer and cannot meet real-time requirements. In contrast, YOLOv8-s requires only 3 ms per tile. After incorporating the overhead of tiling, result merging, and non-maximum suppression (NMS), the complete end-to-end pipeline achieves a latency of 0.359 s (approximately 2.78 FPS), thereby meeting the real-time operational target of the system.

Requirement for Balancing Detection Accuracy and Computational Complexity:The mAP@50:95 score of YOLOv8-s reaches 44.9%, enabling effective identification of multi-scale targets, ranging from 10-pixel small fishing boats to 600-pixel large cargo ships. This provides a solid foundation for further enhancing extreme-scale adaptability using the MSF module and satisfies advanced accuracy requirements. In terms of computational footprint, YOLOv8-s, with 11.2M parameters and 28.6 GFLOPs, is well suited to the resource constraints of the RK3588 embedded system. Its efficiency provides sufficient computational headroom for lightweight optimization within the GSF-Neck module, ensuring hardware compatibility and stable performance on the embedded platform.

## 4. The Optimized YOLOv8-Based Model

### 4.1. Overall Network Structure

**Figure 3:**
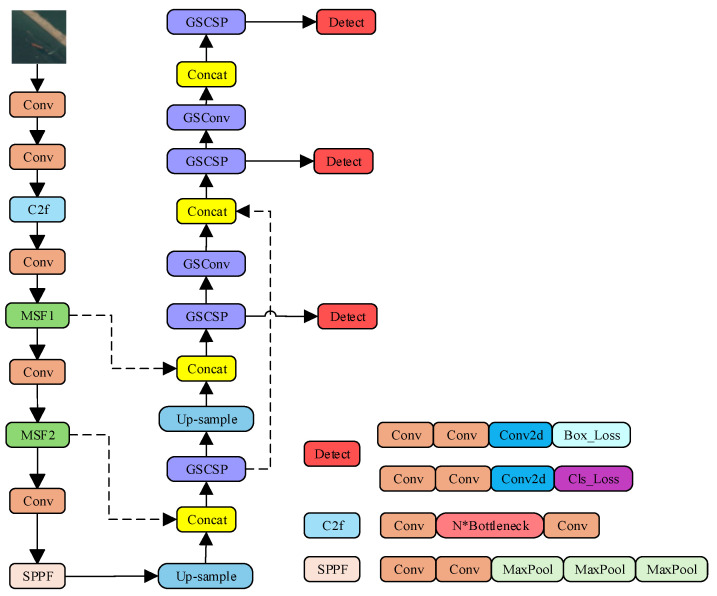
Overall structure of the network.

A two-stage feature extraction optimization strategy is proposed to address the dual challenges in ship detection from high-altitude, large-format aerial images: (i) substantial intra-image scale variation (from 20-pixel small boats to 600-pixel large vessels) and (ii) the requirement for high computational efficiency when processing numerous image tiles in each frame. The two-stage optimization approach addresses these issues through complementary design stages. First, the newly proposed MSF module with a parallel multi-branch architecture is integrated into the backbone to enhance multi-level feature extraction capability. Second, the neck network is redesigned with the GSF-Neck to achieve efficient feature fusion while reducing computational complexity. This two-stage design preserves high feature quality and computational efficiency, making it suitable for real-time object detection and adaptable for embedded deployment. The overall structure of the network architecture is illustrated in Figure 3 (The asterisk “*” in the figure stands for the multiplication sign “×”).

### 4.2. Multi-Scale Fusion (MSF) Module

To address the significant scale variation of maritime targets and the degraded feature quality caused by interference, a Multi-Scale Fusion (MSF) module is designed within the backbone network. It introduces a parallel multi-branch structure after the C2f module to enable adaptive fusion of multi-scale features.

**Figure 4:**
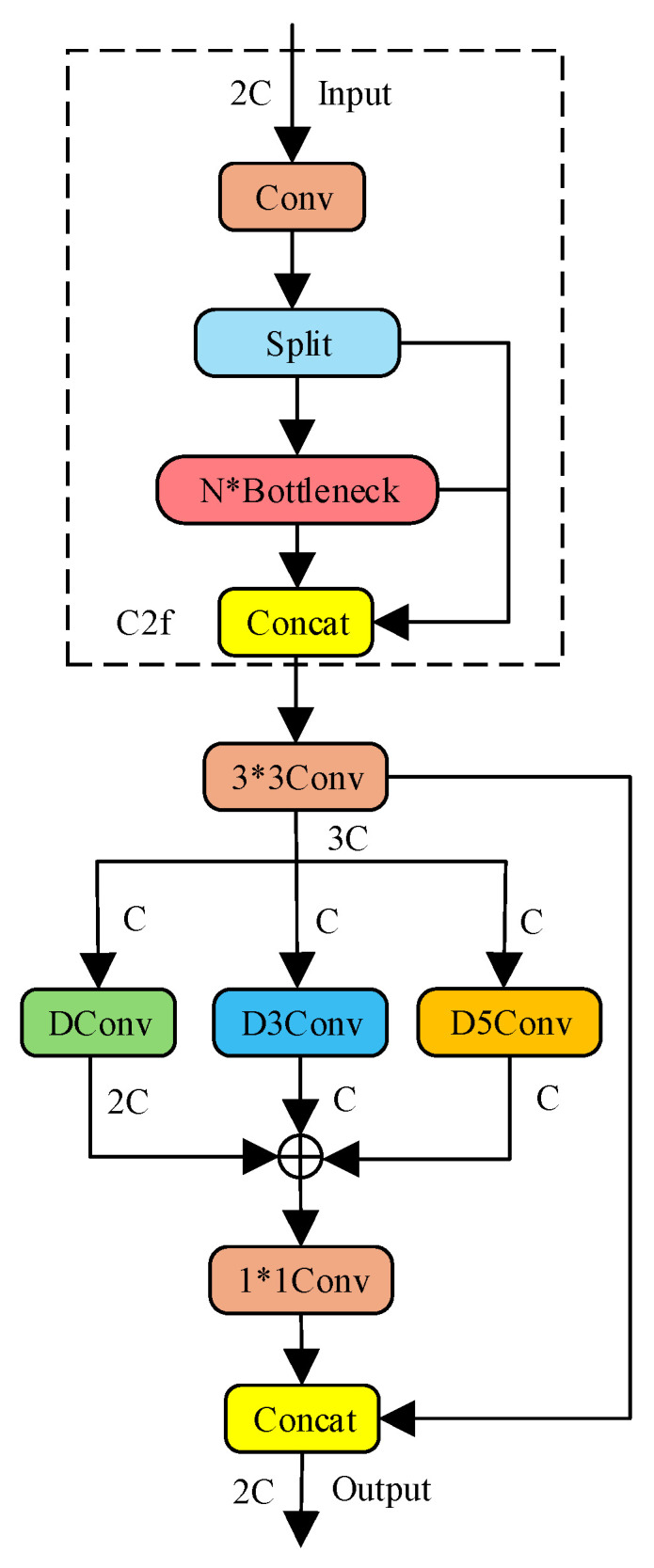
Structure of the MSF module.

The MSF module is deployed at two critical stages within the backbone network, as illustrated in Figure 4 (the asterisk “*” in the figure stands for the multiplication sign “×”). It first employs a bottleneck structure to reduce feature map channels and decrease computational cost. The features are then processed through three parallel branches: one depthwise separable convolution branch and two dilated convolution branches (with dilation rates of 3 and 5), enabling multi-scale feature extraction. Finally, a 1 × 1 convolution is applied to facilitate cross-channel information interaction and feature fusion. It adopts a multi-branch parallel feature extraction structure to effectively capture multi-scale targets. Depthwise separable convolutions are used to extract local fine-grained features, while contextual information at multiple scales is captured using dilated convolutions with different dilation rates. Specifically, parallel dilated convolutions are designed to extract contextual information from small and blurred objects frequently present in large-format aerial images, thereby improving the recognition capability for challenging targets.

The module adopts a multi-level feature optimization mechanism [14], and the algorithm process is described as follows:

The MSF module is an improvement based on the C2f module. Its core idea is to combine residual fusion with multi-scale convolutional branches to enhance feature representation. A preceding C2f module extracts base features and performs residual fusion. It uses a 1 × 1 Conv for convolution. A split operation divides the features into two paths. On one path, N bottlenecks are stacked. Each bottleneck contains a 3 × 3 convolution. The outputs from the bottlenecks are then concatenated with the features from the other path. After concatenation, a 3 × 3 Conv adjusts the number of channels. This prepares the features for the multi-scale branches.

The multi-scale convolutional branches use convolutions with different dilation rates to fuse spatial information at various scales. The DConv branch uses a 3 × 3 kernel with a dilation rate of 1; the D3Conv branch uses a 3 × 3 kernel with a dilation rate of 3; and the D5Conv branch uses a 3 × 3 kernel with a dilation rate of 5. These three branches capture small, medium, and large spatial features. This helps the network understand the relationship between a target and its surroundings. The output feature map Fout is obtained by element-wise addition of the feature maps from the three parallel branches. A 1 × 1 convolution then reduces the number of channels. This result is concatenated with the output from a 3 × 3 convolution to form the final feature map. The specific expression is shown in Equation (1):

(1) $$F_{out}=DWConv\left(F_{in}\right)+DConv_{r=3}\left(F_{in}\right)+DConv_{r=5}\left(F_{in}\right)$$

Here, $F_{in}$ denotes the input feature map, and $F_{out}$ denotes the output feature map. $DWConv\left(\cdot \right)$ represents the depthwise separable convolution operation, which extracts local, fine-grained features. $DConv_{r=k}\left(\cdot \right)$ represents the convolution operation with a dilation rate of k (where k=3,5), which captures contextual information at different scales. “+” denotes element-wise addition, which fuses features from the three branches. By using convolutions with different dilation rates, the module covers a wider range of receptive fields. This improves its adaptability to targets of different sizes and enables effective detection of multi-scale maritime targets.

### 4.3. Group-Wise Scale Fusion Neck (GSF-Neck)

The imaging process of maritime vessel targets is highly complex and influenced by multiple factors, including sea state, variations in illumination, and weather conditions, which makes the accurate extraction and characterization of target information from images a challenging task. In maritime vessel images, both vessel morphology and background noise exhibit substantial variability, and traditional image processing methods often fail to satisfy the requirements for high efficiency and high accuracy.

Although YOLOv8 performs well in object detection and its detection head can distinguish targets from the background to a certain extent, multilayer convolutions and a complex detection head are integrated into its network architecture. This design leads to a significant increase in computational complexity and parameter volume, thereby affecting overall processing efficiency and real-time performance.

To address these issues, an optimized YOLOv8 neck network is proposed, which integrates two modules, GSConv and GSCSP, for neck optimization. The proposed optimization scheme aims to maintain high detection accuracy while improving computational efficiency, reducing model complexity, and adapting to the characteristics of complex maritime vessel images.

The GSConv module adopts depthwise separable convolution to substantially reduce computational cost. A subsequent channel shuffle operation facilitates cross-channel information exchange, compensating for potential performance degradation and ensuring rich feature representation.

**Figure 5:**
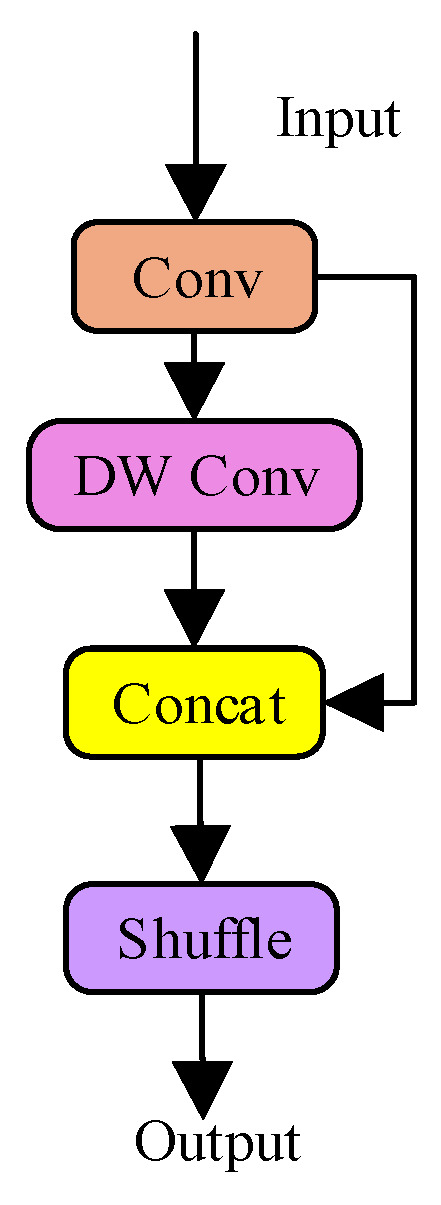
Structure of GSConv.

**Figure 6:**
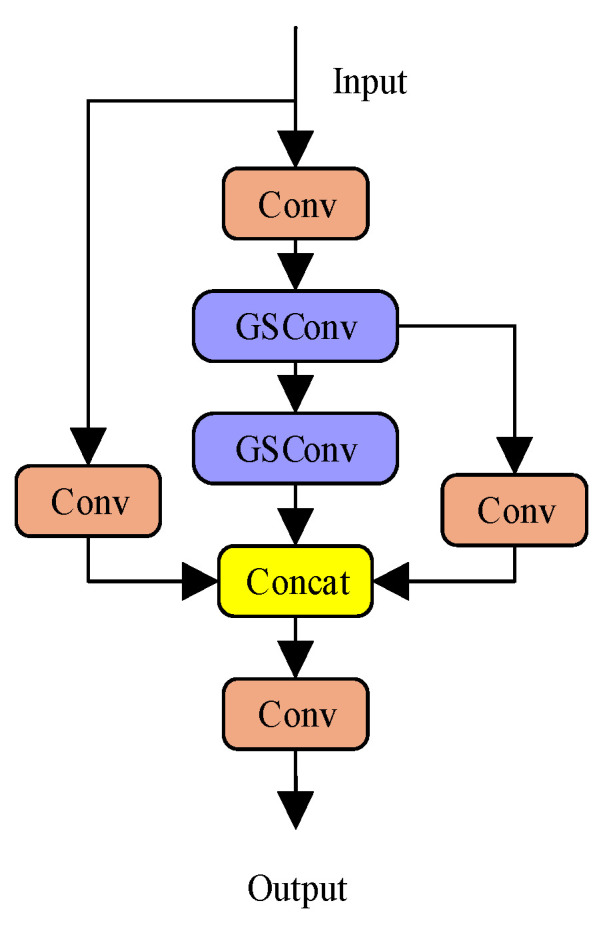
Structure of GSCSP.

The GSCSP module employs a split-and-merge strategy, in which different portions of feature maps are processed through separate paths. This mechanism promotes feature reuse, diversifies gradient propagation, and enhances feature representation under a lower computational burden. The structures of GSConv and GSCSP are illustrated in Figure 5 and Figure 6, respectively.

**Figure 7:**
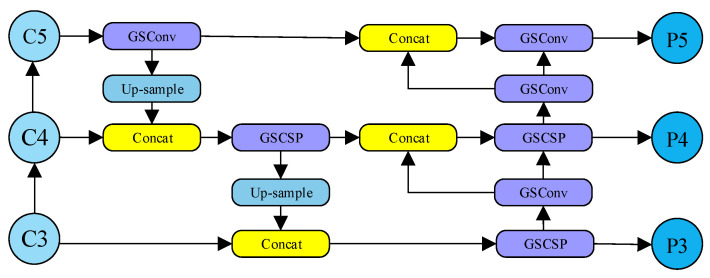
GSF-Neck diagram of grouped expanded neck structure.

The overall GSF-Neck structure is shown in Figure 7. It works as follows:

- Upsampling and Concatenation: Feature maps are upsampled to a higher spatial resolution and concatenated based on feature maps from earlier layers. This preserves fine-grained, low-level detail information at high resolution.
- Efficient Processing and Fusion: The concatenated features are processed through GSConv and GSCSP modules. Their outputs are further fused with features obtained from other paths, forming a comprehensive and rich feature representation.
- Multi-Scale Detection: The final fused multi-scale features are passed to the detection head for predicting object locations and classes.

This design allows the network to accurately localize and identify marine vessels at different scales. It maintains high computational efficiency and exhibits strong robustness under complex background conditions.

The primary reason why GSF-Neck significantly reduces model complexity lies in its replacement of conventional standard convolutions with GSConv, combined with the efficient topology of the GSCSP module. The associated mathematical principles are described below.

During convolution operations, given the input feature map size, output channel number, and convolution kernel size, the FLOPs [15] of standard convolution can be calculated using Equation (2):

(2) $$FLOPs_{std}=H\times W\times C_{in}\times C_{out}\times K^{2}$$

Standard convolution: $H\times W\times C_{in}$; kernel size: K; number of output channels: $C_{out}$.

GSConv decomposes a standard convolution into depthwise separable convolution and pointwise convolution (1 × 1 convolution), with optional channel shuffling. The corresponding FLOPs are calculated as shown in Equation (3):

(3) $$FLOPs_{GSConv}\approx H\times W\times \left(C_{in}\times K^{2}+C_{in}\times C_{out}\right)$$

The ratio between the two is expressed in Equation (4):

(4) $$\frac{FLOPs_{GSConv}}{FLOPs_{std}}\approx \frac{1}{C_{out}}+\frac{1}{K^{2}}$$

When $C_{out}$ is large, 1/$C_{out}$ approaches 0. At this point, the computational load of GSConv is primarily determined by 1/*K*^2^ (for instance, when *K* = 3, the computational cost is only 1/9 that of standard convolution), achieving significant computational savings.

In summary, the proposed GSF-Neck substantially reduces model complexity while improving computational efficiency and preserving strong feature extraction capability. Through effective multi-level feature fusion, it is well adapted for complex detection tasks. Moreover, it facilitates model training and inference, reduces computational resource consumption, and demonstrates strong suitability for real-time applications and large-scale data processing, particularly in maritime surveillance and remote sensing scenarios.

### 4.4. End-to-End Processing Pipeline of Ultra-High-Resolution Large-Format Images

To satisfy the real-time processing requirements of ultra-high-resolution large-format images, an end-to-end pipeline is designed based on the strategy of “overlapping tiling, cross-tile fusion, and parallel inference,” ensuring a balance between processing accuracy and efficiency.

To prevent truncation of cross-boundary objects, a fixed-size overlapping tiling scheme is adopted. Key parameters are determined through quantitative formulas, with the tiling stride and between-tile overlap set to 640 and 256 pixels, respectively. In this manner, medium-sized objects can be fully contained within two tiles. Each image is divided into 330 tiles, enabling comprehensive capture of cross-boundary objects. To align with the hardware capabilities of the RK3588 embedded system, a batch-based parallel inference strategy is implemented. Experimental results indicate that the end-to-end latency reaches about 2.78 FPS, meeting the real-time processing requirement of ≥2 FPS.

To address cross-tile object fusion, the WBF algorithm [16] is applied to resolve object splitting across adjacent tiles. For a single object detected in multiple neighboring tiles, K partial bounding boxes {*B*_1_, *B*_2_, …, *B_k_*} are fused into a complete bounding box using confidence-weighted integration, as shown in Equation (5):

(5) $$x_{min}=\frac{\sum_{i=1}^{k}Conf\left(B_{i}\right)\times x_{min}\left(B_{i}\right)}{\sum_{i=1}^{k}Conf\left(B_{i}\right)}\;y_{min}=\frac{\sum_{i=1}^{k}Conf\left(B_{i}\right)\times y_{min}\left(B_{i}\right)}{\sum_{i=1}^{k}Conf\left(B_{i}\right)}\;x_{max}=\frac{\sum_{i=1}^{k}Conf\left(B_{i}\right)\times x_{max}\left(B_{i}\right)}{\sum_{i=1}^{k}Conf\left(B_{i}\right)}\;y_{max}=\frac{\sum_{i=1}^{k}Conf\left(B_{i}\right)\times y_{max}\left(B_{i}\right)}{\sum_{i=1}^{k}Conf\left(B_{i}\right)}$$

Here, *Conf*(*B_i_*) denotes the confidence score of the *i*-th partial bounding box, while *x_min_*(*B_i_*), *y_min_*(*B_i_*), *x_max_*(*B_i_*), and *y_max_*(*B_i_*) represent its left, bottom, right, and top boundary coordinates, respectively. This formulation accurately reconstructs the integrated cross-tile object contour through confidence-weighted averaging. In addition, a 0.5 IoU threshold is employed to eliminate duplicate detections, thereby ensuring that the fused bounding boxes are both accurate and unique.

## 5. Experimental Results and Analysis

### 5.1. Dataset and Experimental Setup

#### 5.1.1. Dataset Description and Collection Challenges

To validate the effectiveness of the proposed model, a dedicated visible-light grayscale aerial ship dataset comprising 50,454 high-resolution wide-format images was constructed, containing 232,192 annotated ship instances. The dataset was collected using an airborne reconnaissance camera and consists of grayscale images, consistent with common maritime surveillance practice that prioritizes structural details and target contours over color information. These images were acquired during coastal aerial surveys under different daylight conditions (sunny and cloudy) to enhance model robustness. The dataset includes various ship types, such as speedboats, fishing boats, and cargo ships, with substantial scale variation ranging from 10-pixel small fishing boats to 600-pixel large cargo vessels.

A waterborne vessel with a clearly visible hull is defined as a “ship” instance. Superstructures, cranes, and masts were included when connected to the hull, whereas shadows, wakes, and isolated ship components were excluded. For occluded cases, full bounding box estimation was performed when the clear hull area exceeded 50%, while heavily occluded instances were omitted separately.

The dataset was divided into a training set (70%, 35,318 images), a validation set (15%, 7568 images), and a test set (15%, 7568 images) to ensure that no data leakage occurs among subsets.

Annotation Challenges:

- Images from 5000-m altitude often contained cloud occlusions, which were intentionally included to improve model resilience.
- Sun glint and wave reflections resulted in false textures, which were challenging for annotation.
- Extremely small distant vessels demanded high annotation precision.
- Class imbalance between large ships and small fishing boats required meticulous handling.

Annotation Methodology:

- Altitude: 80% at 5000 m; 20% between 3000 and 7000 m.
- Sea State: calm (40%), moderate (40%), and rough (20%) based on wave pattern classification.
- Viewing Angle: nadir (70%), off-nadir up to 30° (30%).
- Time of Day: 8:00–18:00 local time, covering varied sun angles.

**Figure 8:**
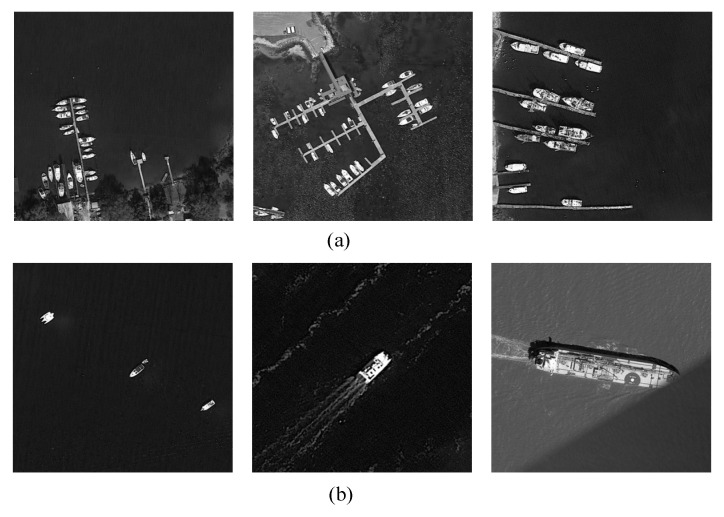
Example images from the dataset (showing both (**a**) dense and (**b**) sparse ship distributions).

Annotations were conducted by three expert annotators using annotation tool, following strict guidelines in which bounding boxes tightly enclosed visible hull regions while excluding wakes and shadows. Cross-validation procedures were implemented to ensure annotation quality and consistency. Examples of dense and sparse ship distributions within the dataset are presented in Figure 8.

#### 5.1.2. Experimental Environment Configuration

- Hardware: Windows 10 workstation with NVIDIA RTX 4090D GPU (24 GB VRAM) and 64 GB DDR5 RAM;
- Software: PyTorch 1.10.0, Python 3.8, and CUDA 11.3;
- Training Parameters: Input resolution 640 × 640, batch size 4, 300 epochs, Adam optimizer (initial LR = 0.01, weight decay = 5 × 10^−4^), cosine annealing scheduler (min LR = 0.002).

### 5.2. Target Detection Evaluation Metrics

Employed standard object detection metrics:

- mAP@0.5: mean average precision at IoU threshold 0.5.

The mean average precision (mAP) [17] is calculated as the area under the precision–recall curve for each class and then averaged across all classes, as shown in Equation (6):

(6) $$mAP=\frac{1}{N}\sum_{i=1}^{N}\int_{0}^{1}P_{i}(R_{i})dR_{i}$$

where P_i_ and R_i_ denote the precision and recall for class i, and N represents the number of classes. This metric comprehensively reflects the detector’s performance across different recall levels.

- mAP@0.5:0.95: mAP averaged over IoU thresholds from 0.5 to 0.95 (step 0.05).
- Params: Number of trainable parameters, indicating model size.
- FLOPs: floating-point operations for one forward pass, measuring computational complexity.
- FPS: frames processed per second, measuring real-time performance.

### 5.3. Ablation Experimental Results and Analysis

Ablation experiments were performed on the maritime vessel dataset using YOLOv8s as the baseline. The results are presented in Table 3.

Both proposed modules enhance model performance. The complete model (V3) increases mAP@0.5 by 1.4% and mAP@0.5:0.95 by 1.44% compared with the baseline. Although FPS decreases slightly relative to the baseline, the notable improvement in accuracy indicates an effective trade-off, substantially enhancing overall detection performance.

The impact of inserting the MSF module at different backbone stages (MSF1 and MSF2) was further investigated. Table 4 indicates that simultaneously integrating both MSF1 and MSF2 achieves the highest accuracy (94.55% mAP@0.5), confirming the importance of multi-scale feature enhancement at multiple network depths.

### 5.4. Comparative Experiment Results and Analyses

The proposed method was compared with several state-of-the-art detectors on the maritime vessel dataset, as shown in Table 5. The proposed method achieved the highest mAP@0.5 (94.55%) among all compared detectors. It significantly outperformed two-stage methods such as Faster R-CNN in both accuracy and speed and surpassed other YOLO variants, demonstrating *a* superior balance between accuracy and speed for this specific task.

### 5.5. Visualization and Per-Class Analysis

**Figure 9:**
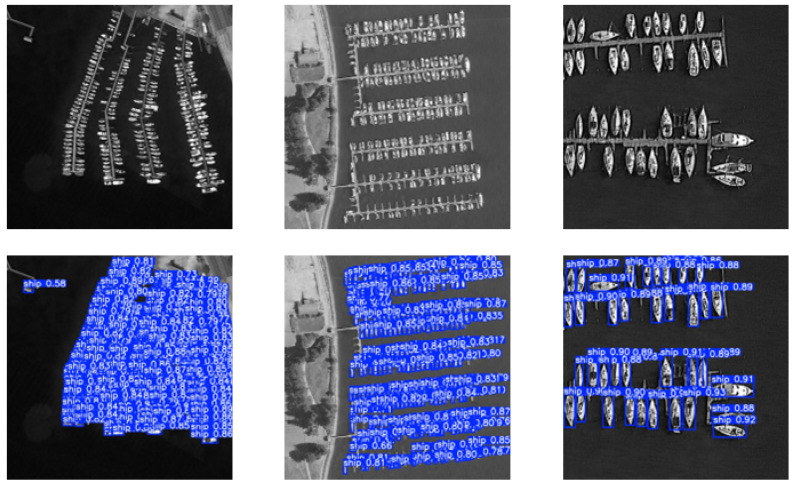
Effect of dense vessel detection.

**Figure 10:**
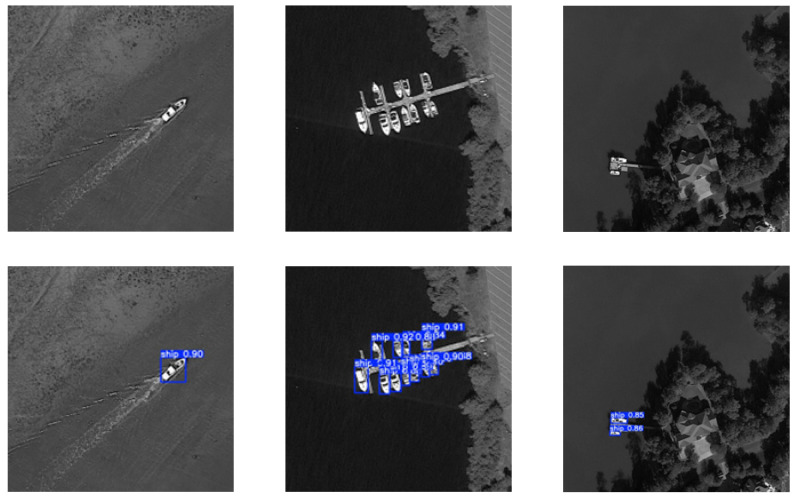
Sparse vessel detection effect.

In order to more intuitively demonstrate the effectiveness of the improved YOLOv8 algorithm in target detection, representative images from the test set were selected for visualization experiments. As shown in Figure 9 and Figure 10, the scenes cover various maritime vessel scenarios, including dense and sparse ship distributions, enabling a comprehensive evaluation of algorithm performance.

Figure 9 shows the detection results in a scene with dense vessel distribution. The proposed model accurately detected and localized multiple closely spaced ships, demonstrating robustness in crowded environments.

Figure 10 illustrates the detection results in a scene with sparse vessel distribution and challenging background conditions (wave reflections and shoreline). The model successfully identified isolated ships while minimizing false positives caused by background interference.

A per-class analysis (Table 6) indicates that the most substantial improvement is observed for “fishing boat,” which is typically the smallest and most challenging category. This finding highlights the enhanced capability of the proposed model in small-object detection, directly benefiting from the multi-scale design.

### 5.6. Model Deployment

The trained improved YOLOv8 model was successfully converted to the RKNN format using toolkit2 (version 2.0.0) and deployed on the RK3588 embedded platform. Post-deployment testing verified the model’s operational stability and enabled memory and performance profiling on the target hardware, demonstrating the practical feasibility of the lightweight design for real-world embedded applications.

### 5.7. Aerial Photography Test Results Analysis

**Figure 11:**
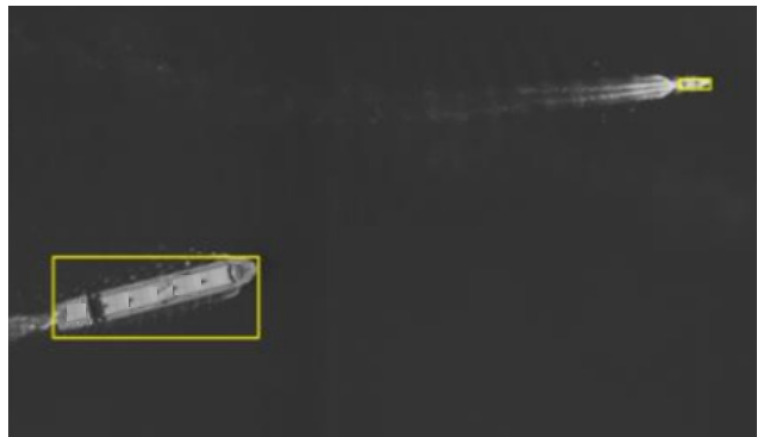
Real-time detection image of maritime targets.

A UAV was employed to carry the platform for capturing real sea surface scenes, enabling real-time target detection and output of target location information. The platform outputs single-image data volumes of 300 MB with a photo cycle of 0.45 s. A total of 3850 images were captured, including 200 images containing vessel targets, of which 179 were correctly detected, resulting in a detection rate of 89.5%. A representative sea target image is shown in Figure 11. Two vessel targets were detected in the aerial image. Target 1 is annotated with the following coordinates: top-left X: 8584; top-left Y: 1234; bottom-right X: 8617; bottom-right Y: 1249. Target 2 is annotated with the following coordinates: top-left X: 4752; top-left Y: 284; bottom-right X: 4795; bottom-right Y: 354.

## 6. Discussion

### 6.1. Interpretation of Key Findings

The ablation study (Table 3) confirms that both the Multi-Scale Fusion (MSF) module and the Group-Wise Scale Fusion Neck (GSF-Neck) improve model performance. The parallel multi-branch design of the MSF module effectively addresses the challenge of extreme scale variation in maritime targets. By applying depthwise separable convolutions to capture local features and dilated convolutions to incorporate multi-scale contextual information, detection performance for both large ships and small vessels is enhanced, as reflected by consistent improvements in mAP metrics when the module is integrated (V1 and V3 vs. baseline and V2).

Simultaneously, the GSF-Neck optimizes the model for embedded deployment. Through the use of GSConv and GSCSP modules, parameters and FLOPs are significantly reduced while maintaining or slightly improving accuracy. This indicates efficient information flow and feature fusion, reducing redundancy without compromising feature quality. In particular, the channel shuffle mechanism within GSConv effectively enhances discrimination between ships and complex backgrounds such as waves.

The integration of both modules in V3 yields the highest accuracy (94.55% mAP@0.5), suggesting their complementary roles: MSF provides enriched multi-scale feature representation, whereas GSF-Neck ensures efficient feature processing.

### 6.2. Comparison with Current Methods

As shown in Table 5, the proposed method demonstrates superior performance in terms of both accuracy and efficiency compared with existing detectors. It achieves higher accuracy and faster inference than two-stage detectors such as Faster R-CNN, consistent with the inherent advantages of single-stage frameworks. Moreover, it outperforms other YOLO variants (YOLOv5, YOLOv8, and YOLOX) while maintaining moderate computational cost and competitive model size. This balance is critical for deployment on resource-constrained platforms such as RK3588. Figure 10 and Figure 11 further validate the robust performance under diverse scene conditions.

### 6.3. Limitations and Future Work

Despite these promising results, certain limitations remain. The dataset is restricted to visible-spectrum daytime imagery, although it has demonstrated effectiveness. Model performance under multi-spectral data or low-visibility conditions (fog, night, or rain) has not yet been evaluated. Although a processing speed of 2 FPS is achieved for individual large images, this may be insufficient for high-frequency tracking applications. Therefore, further analysis of thermal performance and energy consumption on the RK3588 platform is necessary to evaluate long-term embedded operation.

Several failure cases were identified during qualitative analysis: (1) missed or merged detections for extremely small and dense targets (<20 pixels), indicating potential resolution limitations; (2) reduced performance under unusual viewpoints or severe occlusion; (3) occasional false positives under intense sun glint, suggesting sensitivity to extreme illumination conditions.

Future work should focus on (1) expanding the dataset to include multi-temporal, multi-weather, and multi-spectral imagery; (2) applying advanced model compression techniques (quantization, pruning) to further improve inference speed; (3) conducting comprehensive thermal and energy profiling on the embedded platform; and (4) integrating tracking algorithms for video-based analysis.

### 6.4. Theoretical and Practical Implications

This study validates key design principles for effective computer vision systems under resource constraints. The MSF module demonstrates that parallel and explicit multi-scale feature extraction is essential for handling extreme object size variation, which is common in remote sensing scenarios. The GSF-Neck represents a co-design strategy that combines efficient operators (GSConv) with structural patterns (GSCSP) to achieve an improved accuracy–efficiency trade-off. It provides a valuable framework for developing vision models constrained by computational complexity, offering both a high-performance ship detector and a reproducible design paradigm for addressing similar challenges.

## 7. Conclusions

This study proposes an optimized YOLOv8-based ship detection algorithm to address extreme scale variation and complex backgrounds in large-format aerial imagery. The approach introduces two key structural innovations: the MSF module integrated into the backbone and the GSF-Neck.

The MSF module employs a parallel multi-branch structure with dilated and depthwise separable convolutions, significantly enhancing feature extraction and fusion capabilities across extreme scales, from small boats to large vessels. Meanwhile, the GSF-Neck, incorporating GSCSP and GSConv modules, efficiently fuses features and facilitates cross-channel information interaction, substantially reducing model parameters and computational complexity while preserving strong feature representation.

Experimental results on the dedicated aerial maritime dataset demonstrate clear performance improvements. The final model achieves a state-of-the-art mAP@0.5 of 94.55%, outperforming existing detectors such as YOLOv5, Faster R-CNN, and the original YOLOv8 while maintaining a computational footprint suitable for embedded deployment. Notably, the most significant accuracy gains are observed for small and challenging targets, particularly fishing boats. Successful deployment and operation on the RK3588 embedded platform further confirm the practical applicability of the proposed method in real-world scenarios.

This work provides a valuable design framework for balancing multi-level feature representation and computational efficiency under resource-constrained conditions. Future research will focus on enhancing model robustness under all-weather conditions and further improving inference speed for high-frequency tracking applications.

## Figures and Tables

**Table 1:** Design parameters of the photoelectric imaging system.

Parameter	Photoelectric Imaging Module Indicators
System form	Double lens splicing
Focal length (mm)	126
Field of view (°)	62
Waveband (μm)	0.486~0.656
Image resolution GSD (m)	0.19
Ground width (km)	≥6
Detector pixel (μm)	4.6
Number of spliced detectors	4

**Table 2:** Performance of different models for target detection.

Model	Size (Pixels)	mAP@50:95	Speed CPU ONNX (ms)	Speed A100 TensorRT (ms)	Params (M)	FLOPs (B)
YOLO8n	640	37.3	80.4	0.99	3.2	8.7
YOLO8s	640	44.9	128.4	1.20	11.2	28.6
YOLO8m	640	50.2	234.7	1.83	25.9	78.9
YOLO8l	640	52.9	375.2	2.39	43.7	165.2
YOLO8x	640	53.9	479.1	3.53	68.2	257.8

**Table 3:** Ablation study of the proposed MSF and GSF-Neck modules. V1: baseline + MSF; V2: baseline + GSF-Neck; V3: baseline + MSF + GSF-Neck (proposed).

Methods	MSF	GSF-Neck	mAP@0.5/%	mAP@0.5:0.95/%	Param (M)	FLOPs (G)	FPS
Baseline	-	-	93.15	74.12	11.2	28.6	93.2
V1	√	-	93.34	74.18	12.1	29.4	88.5
V2	-	√	93.39	74.91	10.8	25.9	95.7
V3	√	√	94.55	75.56	11.5	26.7	86.6

Note: Params and FLOPs for V2 and V3 are estimated based on the architectural modifications described in Section 4.

**Table 4:** Performance evaluation of different MSF module configurations.

Method	MSF1	MSF2	Param (M)	FLOPs (G)	mAP@0.5/%	mAP@0.5:0.95/%	Recall/%	FPS
Baseline	-	-	11.2	28.6	93.15	74.12	90.12	93.2
1	√	-	12.0	29.1	93.49	74.23	90.24	82.3
2	-	√	11.8	29.3	93.74	74.37	90.51	85.7
3	√	√	12.2	29.7	94.55	75.56	91.17	88.5

**Table 5:** Performance comparison with state-of-the-art detectors.

Methods	mAP@0.5 (%)	FPS
Faster-RCNN	87.32	25.2
Retina Net	81.16	42.5
YOLOv5	88.32	78.6
YOLOv8	92.44	82.3
YOLOX	92.76	65.4
Proposed method	94.55	86.6

**Table 6:** Per-class average precision (AP@0.5) analysis.

Vessel Category	Baseline AP (%)	Proposed Model AP (%)
Cargo Ship	96.8	97.1
Passenger Ship	95.2	95.5
Fishing Boat	82.4	86.6
Speedboat	89.7	91.5
Overall (mAP)	93.15	94.55

## Data Availability

The original contributions presented in this study are included in the article. Further inquiries can be directed to the corresponding authors.
